# Temperature Evolution of Crystal Structure, Ferroelectricity and Ionic Conductivity of Ca_9_La(VO_4_)_7_

**DOI:** 10.3390/molecules31060984

**Published:** 2026-03-15

**Authors:** Oksana V. Baryshnikova, Bogdan I. Lazoryak, Vladimir A. Morozov, Sergey Yu. Stefanovich, Alexander V. Mosunov, Eldar M. Gallyamov, Sergey M. Aksenov, Dina V. Deyneko

**Affiliations:** 1 Faculty of Chemistry, MSU-BIT University, Shenzhen 518172, China; 2 Department of Chemistry, Lomonosov Moscow State University, 119991 Moscow, Russia; 3 Laboratory of Arctic Mineralogy and Material Sciences, Kola Science Centre, Russian Academy of Sciences, 14 Fersman Str., 184209 Apatity, Russia

**Keywords:** orthovanadates, calcium, lanthanum, crystal structure, non-linear optical activity, ferroelectric, ionic conductivity, phase transition

## Abstract

The properties of a Ca_9_La(VO_4_)_7_ single crystal were studied using dielectric spectroscopy and second-harmonic generation. The crystal structure of Ca_9_La(VO_4_)_7_ grown using the Czochralski technique was refined using single-crystal data. The distribution of Ca^2+^ and La^3+^ cations over structural positions was determined. The crystal structure refinement results were compared with those obtained previously from powder X-ray diffraction data. It was shown that the refinement carried out using two different data sets leads to approximately the same results for the distances in the polyhedra, but their distortion is significantly less in the case of using single-crystal data for calculation. Dielectric properties and conductivity measurements were performed on polished single-crystal wafers cut parallel and perpendicular to the c axis. Second-harmonic generation and dielectric temperature measurements revealed the presence of a reversible ferroelectric first-order phase transition at about 1224 K from the ferroelectric β-phase (space group *R*3*c*) to the paraelectric β′-phase. The ferroelectric–paraelectric phase transition is accompanied by a complex structural rearrangement, including a 60° rotation of the V1O_4_ tetrahedron, as well as slight displacements of the Ca^2+^ and La^3+^ cations. It has been shown that the conductivity differs only slightly along the polar axis and perpendicular to it. Above the phase transition temperature, the activation energy of the conductivity is the same for all directions, E_a_~1.2 eV. The influence of composition on the phase transition temperature and the formation of ferroelectric and nonlinear optical properties is discussed.

## 1. Introduction

Phosphate and vanadate compounds crystallizing in the β-Ca_3_(PO_4_)_2_-type (β-TCP) structure (space group (SG) *R*3*c*, Z = 21) [1] are characterized by high chemical and thermal stability, as well as an appropriate band gap for optoelectronic applications. Among these, Ca_3_(VO_4_)_2_—which adopts a β-TCP-related structure (*R*3*c*, Z = 21; also reported as Ca_10.5_(VO_4_)_7_, Z = 6)—has been extensively employed as a host matrix for rare earth element (*REE*) cations to generate photoluminescence from *REE*^3+^ ions [2,3,4,5]. Depending on the specific *REE* ion (*REE*^3+^ or *REE*^2+^), these doped materials can generate emission across the visible [6,7,8,9] and near-infrared spectral regions [10,11]. Moreover, certain β-TCP-type vanadates lack a center of symmetry and demonstrate nonlinear optical properties [12,13,14], making them promising candidates for multifunctional materials through strategic cationic substitutions.

Among these, Ca_9_La(VO_4_)_7_ (CLVO) crystallizes in the β-TCP-type structure [15,16] and has been extensively investigated as a host for luminescent materials. Although the undoped CLVO matrix does not exhibit significant photoluminescence, it readily accommodates activator ions, particularly *REE* cations, into its crystal lattice. Numerous studies have characterized doped CLVO:*REE*, including its luminescence [6,8,9,17,18,19,20,21,22], laser performance [11,12,13,23,24], nonlinear optical behavior [12,13,14,23], thermal properties [25,26], spectral characteristics [13,21,22], refractive indices [27], ion-conducting properties [28,29], and Raman spectra [30,31]. Further motivation for investigating such vanadates is the possibility of growing them as large single crystals with excellent optical quality [10,11,12,13,23,25,32,33]. As follows from the analysis of the literature, CLVO activated by REE cations exhibits polyfunctional properties and is actively studied. However, data on the dielectric, nonlinear optical, and ion-conducting properties of CLVO are absent in the literature.

In this paper, we investigate the CLVO single crystal grown using the Czochralski method. The structure of CLVO is refined on single-crystal data, and dielectric, nonlinear optical, and ion-conducting properties are studied over a wide temperature range. It was demonstrated that CLVO single crystals combine ferroelectric behavior with nearly isotropic ionic conductivity, and fundamental insights into structure-property relationships in β-TCP-type materials are provided. Deeper structural confirmation of all these properties stimulates the development of new multifunctional materials based on CLVO and related compounds.

## 2. Results

### 2.1. SHG and DSC Study

**Figure 1:**
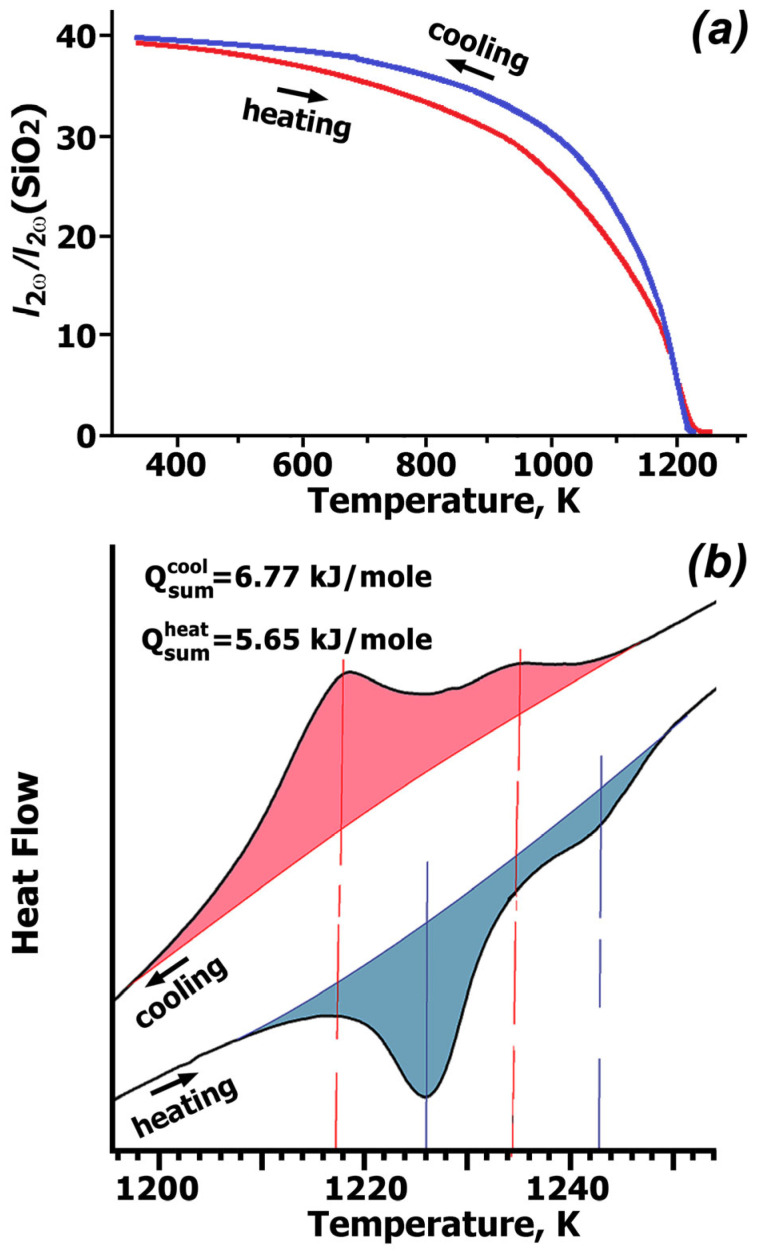
Temperature dependence of the SHG signal (**a**) and heating/cooling DSC curves (**b**) of Ca_9_La(VO_4_)_7_.

Figure 1a shows the temperature dependences of the SHG signal (*I*_2ω_/*I*_2ω_(SiO_2_) upon heating/cooling of CLVO. The presence of the SHG response (I_2ω_/I_2ω_(SiO_2_) = ~40) at room temperature (T_R_) indicates the absence of a symmetry center in this structure, confirming the non-centrosymmetric structure with SG *R*3*c*. Upon heating, the signal regularly decreases and disappears above Curie temperature T_c_ ≈ 1220 K (Figure 1a), thus indicating the appearance of a symmetrical center in the CLVO structure. Upon cooling, the SHG signal is restored with a small temperature hysteresis. This behavior reveals a reversible phase transition: the structure shifts from non-centrosymmetric to centrosymmetric upon heating and reverts to the former upon cooling.

During a first-order phase transition, the SHG signal should change stepwise with temperature, as observed in Ca_9_Fe(PO_4_)_7_ [34]. However, CLVO shows a smoother change in the SHG signal when approaching T_c_, both during heating and cooling (Figure 1). This smooth change indicates a gradual structural transition over a wide temperature range. The SHG signal probably decreases smoothly (heating) or increases smoothly (cooling) due to the smooth ordering (or disordering) of cations and tetrahedrons. The order/disorder in the structure is described in detail in the Section 3.

Figure 1b shows fragments of the DSC heating/cooling curves for CLVO. These curves exhibit two peaks in the temperature range of 1210–1250 K (heating) and 1200–1244 K (cooling). Two endothermic (T_c1_ = 1226 K and T_c2_ = 1243 K) and two exothermic (T_c1_ = 1217 K and T_c2_ = 1234 K) effects on the heating/cooling curves, respectively, indicate the presence of two reversible first-order phase transitions in CLVO. Similar peaks in the DTA curves at 1215 K and 1231 K were also previously observed for CLVO [29]. Two peaks in the DSC curves are apparently associated with a change in symmetry, *R*3*c* → *R*$\bar{3}$c and *R*$\bar{3}$c → $R\bar{3}m$, as in Ca_9_Yb(VO_4_)_7_ [14]. Small values of the thermal effects of the phase transitions upon heating (Q = 5.65 kJ/mol) and cooling (Q = 6.67 kJ/mol) indicate an order–disorder transition in CLVO without a significant change in the chemical bond energy. Similar order–disorder phase transitions were discovered in the ferroelectrics LaBSiO_5_ and LaBGeO_5_, where Q ≈ 0.4–0.7 kJ/mol [35].

### 2.2. Dielectric Properties of Ca_9_La(VO_4_)_7_

**Figure 2:**
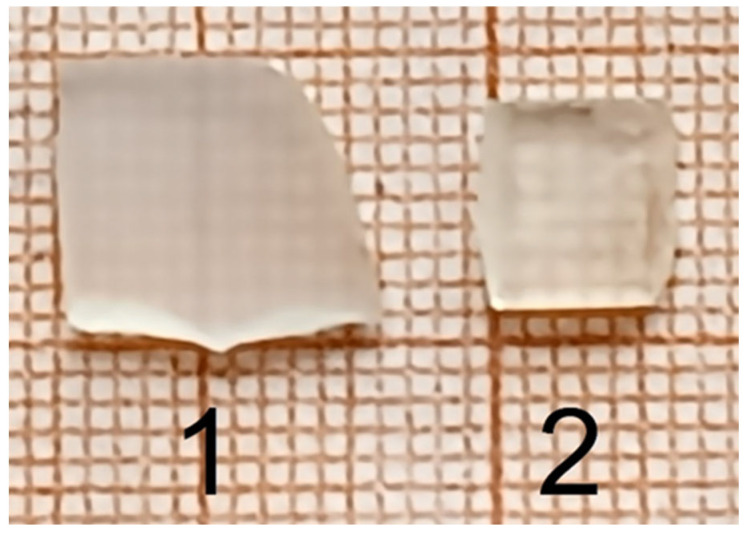
Images of Ca_9_La(VO_4_)_7_ polished crystal wafers cut parallel (1—║) and perpendicular (2—┴) to the *c* axis.

To measure the dielectric properties, two polished crystalline wafers were prepared: one with a 53 mm^2^ (9.3 mm × 5.7 mm) area and 1.55 mm thickness (crystal no. 1, (║)) and one with a 47 mm^2^ area (7.1 mm × 6.6 mm) and 1.45 mm thickness (crystal no. 2, (┴)) (Figure 2).

**Figure 3:**
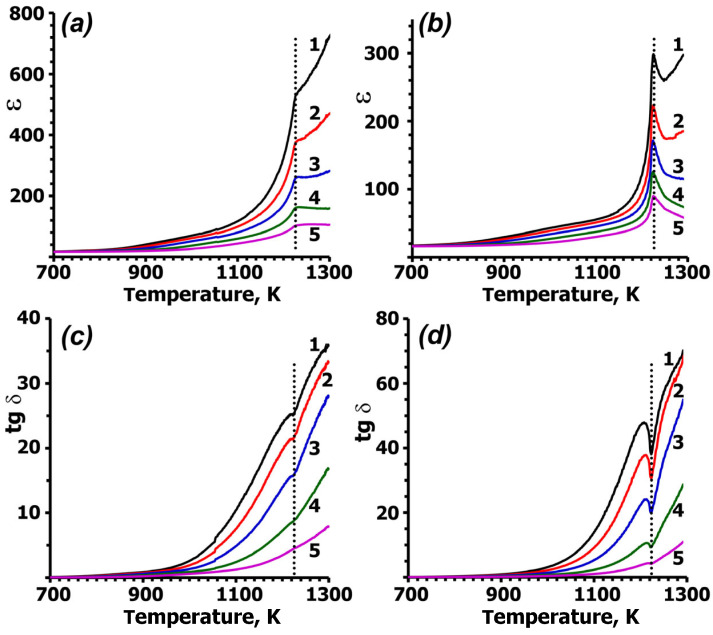
Dielectric permittivity ε (**top**) and dielectric loss tangent tgδ (**bottom**) at frequencies of 30 (1), 50 (2), 100 (3), 300 (4) and 1000 kHz (5) for Ca_9_La(VO_4_)_7_ crystal wafers: 1 (**a**,**c**) and 2 (**b**,**d**).

Figure 3 shows the temperature dependence of the permittivity (ε) and the dielectric loss tangent (tgδ) for the CLVO crystal wafers. All ε(T) curves exhibited a maximum in the range of 1200–1250 K. The position of this maximum was independent of the applied electric field frequency and measurement direction (║ or ┴). The permittivity (ε) decreased with increasing electric field frequency. The CLVO crystal showed higher permittivity along the *c* axis (Figure 3).

The temperature position (T_c_ = 1224 K) of the maximum of peaks on the ε(T) curves approximately coincides with the phase transition temperature found using the SHG and DTA methods, which is T_c_ = 1215 K [29]. The behavior of the ε(T) curves near T_c_ obeys the Curie–Weiss law, confirming a ferroelectric (FE) nature of the phase transition. The tgδ(T) curves show a characteristic minimum (Figure 3), which relates to domain mobility near the phase transition temperature. As the system approaches the phase transition temperature, the domains disappear, reducing their contribution to the electrical conductivity. This disappearance creates a characteristic minimum in the tgδ(T) curve at T_c_. Such a minimum is typical of FE phase transitions. In contrast, antiferroelectrics (AFE) of similar composition, which lack domains, do not exhibit this minimum [36]. These results confirm that CLVO undergoes a reversible FE phase transition at the Curie temperature T_c_ = 1224 K.

**Figure 4:**
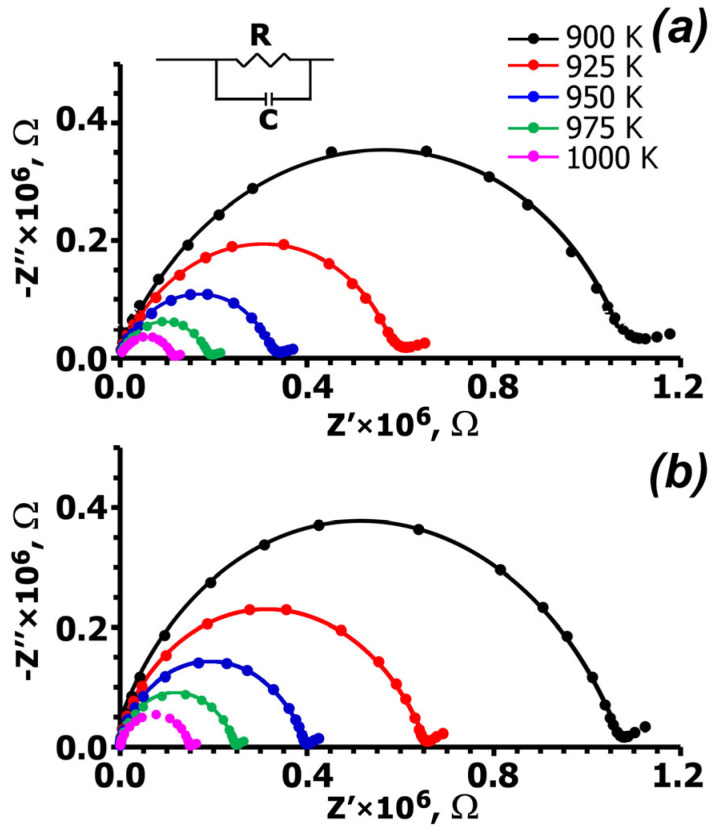
Complex impedance curves of Ca_9_La(VO_4_)_7_ at temperatures of 900, 925, 950, 975 and 1000 K for Ca_9_La(VO_4_)_7_ crystal wafers: 1 (**a**) and 2 (**b**).

**Figure 5:**
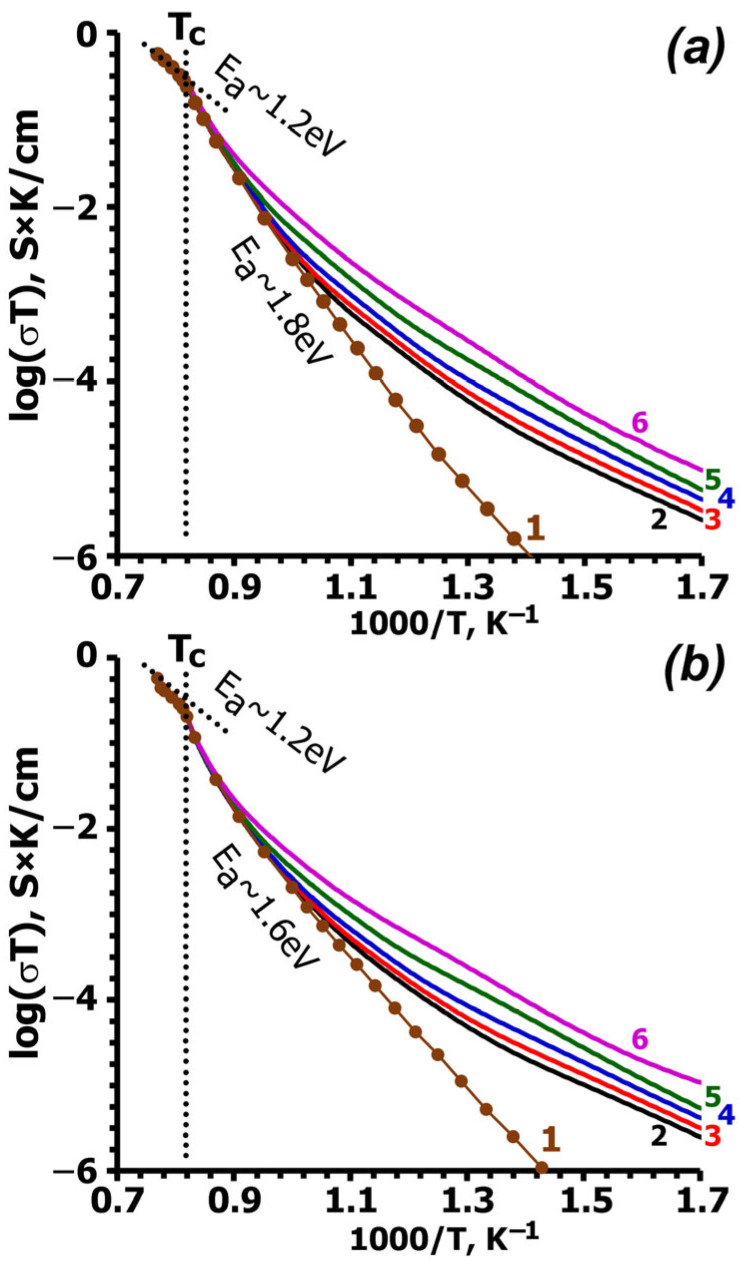
Temperature dependences of the conductivity calculated (1) from the impedance curves and the conductivity at frequencies of 100 Hz (2), 1 kHz (3), 10 kHz (4), 100 kHz (5) and 1 MHz (6) for Ca_9_La(VO_4_)_7_ crystal plates: 1 (**a**) and 2 (**b**).

Cole–Cole diagrams of the complex impedance for single-crystal wafers at various temperatures are shown in Figure 4. The impedance diagram exhibits typical semicircles associated with bulk resistance. The impedance curves were used to calculate the temperature dependence of conductivity (σ), which is independent of crystal orientation (Figure 5).

Figure 5 shows the temperature dependence of conductivity in two crystal directions. The conductivity curves exhibit kinks, the temperature positions of which coincide with the maximum permittivity and the temperature at which the polar phase disappears in the SHG curves. The conductivity values along the two directions in the CLVO differ only slightly, indicating that ion transport in the structure is isotropic. Above the phase transition, the calculated activation energy of conductivity (E_a_~1.2 eV) is the same for both crystal directions. Below the phase transition (around 1200 K), the activation energy differs only slightly between the two directions: E_a_~1.8 eV for crystal wafer 1 and 1.6 eV for crystal wafer 2.

### 2.3. Crystal Structure of Ca_9_La(VO_4_)_7_ Based on Single-Crystal Data

A crystal (0.11 × 0.10 × 0.15 mm) of CLVO was used for single-crystal X-ray data collection. A total of 20,426 reflections within the sphere limited by θ = 30.56° were registered. The details of the experimental data collection process and refinement results are listed in Table 1.

The following trigonal lattice parameters were obtained by least-squares refinement: *a* = 10.9018(2) Å, *c* = 38.1593(6) Å, *γ* = 120°. In accordance with the analysis of systematic absence of reflections, there are two possible space groups, *R*$\bar{3}$c and *R*3*c*, which differ in the presence/absence of the center of symmetry. Based on the SHG data and the |E^2^-1| statistics, we have suggested a non-centrosymmetric space group, *R*3*c* (which is traditional for the β-TCP-related structure). After averaging equivalent reflections, the experimental data set contained 1197 reflections with *I* > 3σ(*I*).

**Figure 6:**
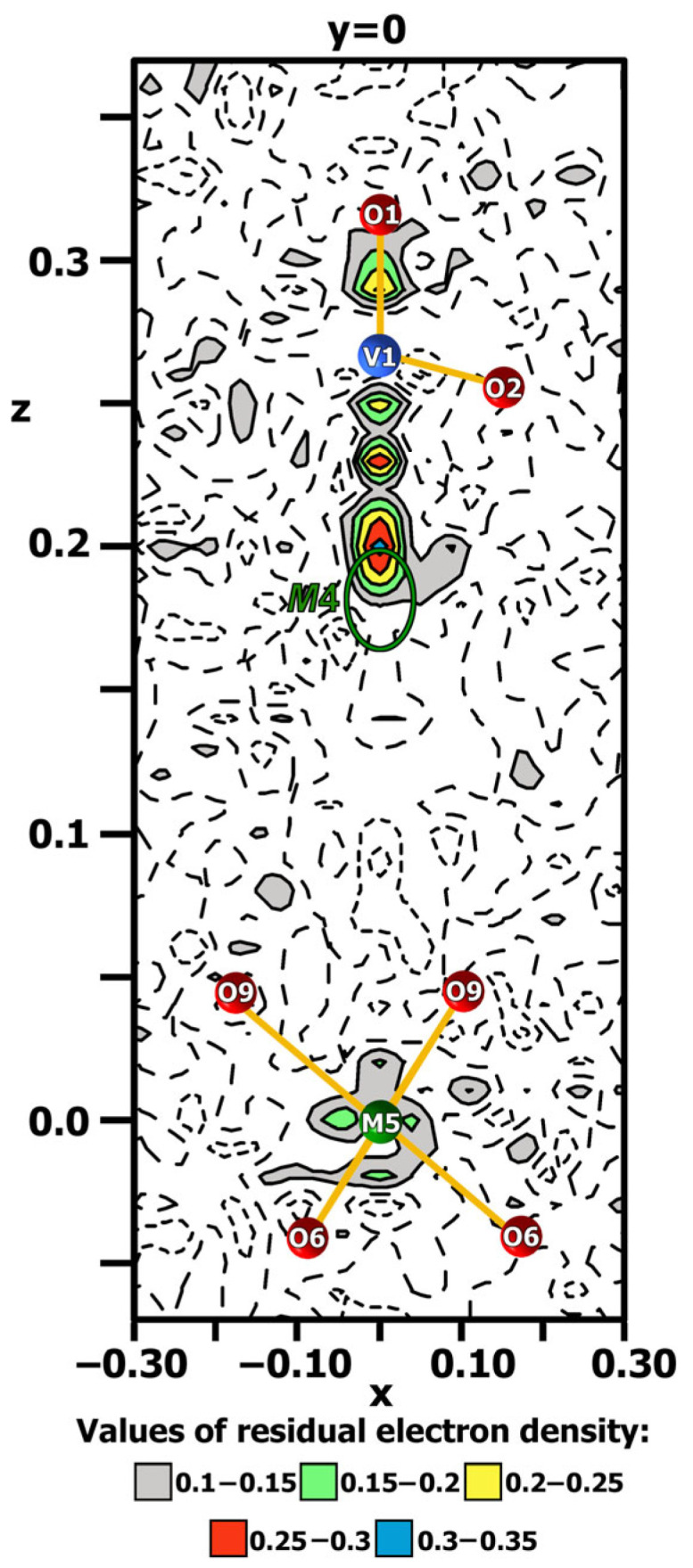
The difference electron density map around the *M*4 and *M*5 positions of the Ca_9_La(VO_4_)_7_ crystal structure. Solid lines indicate positive electron density and dashed lines indicate negative electron density. *M*4 position is shown in the green ellipse. Contour intervals are 0.05 *e* × Å^3^. A color scale for the positive electron density is shown.

**Figure 7:**
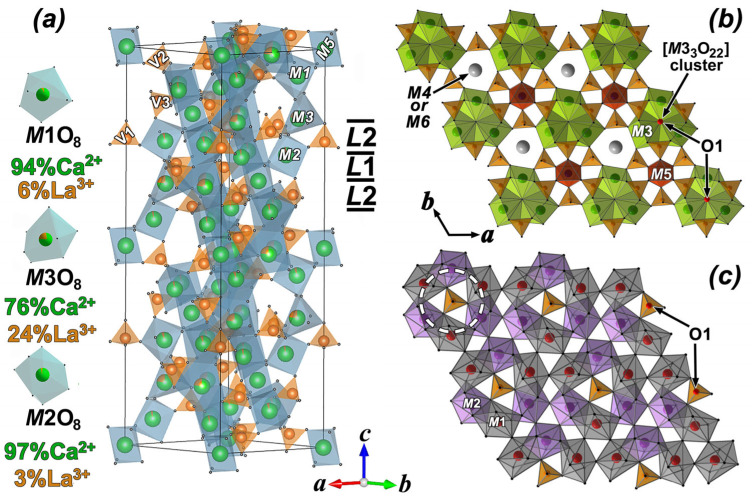
General view of the structure of Ca_9_La(VO_4_)_7_ (**a**), *L*_1_-layer (**b**) and *L*_2_-layer (**c**).

The initial model for CLVO structure refinement was based on the atomic coordinates of the same compound obtained using the Rietveld method [16]. There are six cationic (*M*1–*M*6) sites in β-TCP-related structures. The *M*1–*M*3 (18-*fold*) and *M*5 (6-*fold*) sites are always occupied, while the occupation of the *M*4 (6-*fold*) site can change from 0 to 1, and the *M*6 (6-*fold*) site is usually vacant. According to Belik et al. [16], the La^3+^ and Ca^2+^ cations occupy *M*1–*M*3, and only Ca^2+^ cations occupy *M*5 sites of the CLVO structure. The distribution of La^3+^ and Ca^2+^ cations over the *M*1–*M*3 sites was refined considering their multiplicities, (*M*1–*M*3) = *n*Ca^2+^ + (1 – *n*)La^3+^), with stoichiometric constraints on the overall La/Ca ratio. The positional (*x*; *y*; *z*) parameters and atomic displacement parameters (ADP) of La^3+^ and Ca^2+^ cations over the *M*1–*M*3 sites were refined to be identical. The final refinement cycles converged with *R*_1_ = 1.78, w*R*_2_ = 1.84, and GOOF = 1.14 for all data. Analysis of the difference electron density map [ρ_dif_: (*x*; *y*; *z*)] revealed the absence of additional peaks with noticeable intensity. The highest peak and the deepest minimum in the final residual electron density were equal to 0.32 *e* × Å^3^ and −0.61 *e* × Å^3^, respectively. Figure 6 illustrates difference electron density around the *M*4 site. As one can see, there is no noticeable electron density around the *M*4 and *M*5 sites. These data confirm the chemical composition of the single crystal as Ca_9_La(VO_4_)_7_, and its composition does not change during crystal growth and corresponds to the stoichiometry of the initial batch. These data are of practical importance because they allow the growth of single crystals of a given composition. During single-crystal growth, its stoichiometry could change due to a decrease in lanthanum content and charge compensation due to the incorporation of calcium cations into the *M*4 site. If stoichiometry is disrupted, the crystal composition will be described by the formula Ca_9+2*x*/3_La_1−*x*_(VO_4_)_7_. The properties of such a single crystal will differ significantly from the stoichiometric one (see the Section 3). As a result of the structure refinement, it was established that calcium and lanthanum cations statistically occupy the positions *M*1 = 0.94Ca^2+^ + 0.06La^3+^, *M*2 = 0.97Ca^2+^ + 0.03La^3+^ and *M*3 = 0.76Ca^2+^ + 0.24La^3+^ (Figure 7a, Table S1 of the Supplementary Materials). The *M*5 position is occupied by calcium cations. The high quality of the obtained results and the absence of correlation between pseudo-symmetrical sites confirm the correct choice of the noncentrosymmetric SG.

Table S1 of the Supplementary Materials lists the fractional atomic coordinates, occupancy, site symmetry, and equivalent atomic displacement parameters. Anisotropic atomic displacement parameters are given in Table S2 of the Supplementary Materials. Selected inter-atomic distances and angles in VO_4_^3−^ tetrahedra are given in Table S3 of the Supplementary Materials. The CSD deposition number is 2524264.

## 3. Discussion

Figure 7a shows the general view of the CLVO structure. The structure is constructed from two types of columns elongated along the c-axis. The first column sequentially alternates between tetrahedrons and occupied and unoccupied polyhedra: …-V1O_4_-*M*4O_15_-*M*5O_6_-*M*6O_13_-.… (Figure 7a). The *M*4O_15_ and *M*6O_13_ polyhedra in the CLVO structure are unoccupied, and the *M*5O_6_ polyhedron is occupied by calcium cations. The second column is constructed from tetrahedra and *M*O_8_ polyhedra: …-V2O_4_-V3O_4_-*M*1O_8_-*M*3O_8_-*M*2O_8_-… The *M*1, *M*2, and *M*3 positions are occupied by calcium and lanthanum cations (Figure 7a). VO_4_^3−^ tetrahedra link *M*O_n_ polyhedra into a 3D framework. The 3D framework consists of 12 layers of two types formed by *M*3O_8_ + *M*5O_6_ + V2O_4_ + V3O_4_-polyhedra (*L*_1_-layer) and *M*1O_8_ + *M*2O_8_ + V1O_4_-polyhedra (*L*_2_-layer) alternating along the [001] direction (Figure 7b,c). The *L*_1_-layer contains *M*3O_8_ and *M*5O_6_ polyhedra, which are linked by V2O_4_ and V3O_4_ tetrahedra (Figure 7b). The six-membered cavities in the *L*_1_-layer are formed by the edge of the three *M*3O_8_ and three VO_4_ tetrahedra (Figure 7b), while *M*4 or *M*6 sites (do not occupy in the CLVO structure) are located in these cavities. The *M*3O_9_ polyhedra, linked by common edges, form the [*M*3_3_O_22_] cluster, and adjacent clusters are linked by the VO_4_ tetrahedra edge. The O1 atom is shared by the three *M*3O_9_ polyhedra forming the [*M*3_3_O_22_] cluster. The *L*_2_-layer contains *M*1O_8_ and *M*2O_8_ polyhedra, which form a six-membered ring. The center of each ring is occupied by V1O_4_ tetrahedra (Figure 7c).

The CLVO structure was previously refined using PXRD data [16]. Table 2 lists the comparison of the CLVO structure refined from single-crystal and PXRD data. The lattice parameters and cation site occupancies differ slightly between the two refinements. The average *M*1-O, *M*2-O, *M*3-O and *M*5-O remain virtually unchanged. The difference between two refinements is observed for the polyhedra distortion index and tetrahedral distortion parameters. The following formula was used to estimate the polyhedra distortion index (DI):

(1) $$DI=\;\frac{1}{n}\sum_{i=1}^{n}\frac{\left|l_{i}-l_{av}\right|}{l_{av}},$$

where *n* is the coordination number of central cation, *l*_i_ is the distance from the central cation to the O atom and *l*_av_ is the average bond length. The calculated DI values are presented in Table 2.

In the CLVO structure, the V-O bonds are the strongest. Cations with different charges and sizes within the structure distort the VO_4_^3−^ tetrahedra. The distortion of the tetrahedra is associated with the stretching of the V-O bonds or the bending of the O-V-O bond angles. The distortions of the VO_4_^3−^ tetrahedra were estimated by the change in the distance Δ*d* and the deformation Δα, which characterize the deviations of the V-O distances from the average value and the deviations of the O-V-O angles from the ideal tetrahedral angle (109.5°) (Table 2). The distortion parameters of the VO_4_ tetrahedra were estimated as follows:

(2) ∆d=14∑n=1−4dn−dd2, ∆α=16∑n=1−6αn−αα2,

where *d*_n_ is the individual V-O bond length, *d* is the average V-O bond length, α_n_ is the individual O-V-O bond angle and α is the perfect tetrahedral angle of 109.5°.

According to Table 2, the DI for the *M*2O_8_ and *M*3O_8_ polyhedra differ slightly between the two refinements, while the difference for the *M*1O_8_ polyhedra is more noticeable (approximately 1.4 times). The DI value for *M*5O_6_ calculated from the refinement of the single-crystal data is approximately 4.5 times smaller than the one calculated from the PXRD data. The tetrahedral distortion parameters Δ*d* and Δα show a different trend. The Δ*d* for all VO_4_ and the Δα for the V1O_4_ and V2O_4_ tetrahedra are significantly smaller for the single-crystal data refinement, while the Δα for the V3O_4_ tetrahedron is the same. Thus, the refinement carried out using two different data leads to approximately the same results for the distances in O polyhedra, but their distortion is significantly less in the case of using single-crystal data for the calculation.

For a CLVO single crystal, the calculated σ value at 1200 K does not demonstrate anisotropy and differs slightly along the two directions: (σ = 0.13 × 10^−3^ S/cm for crystal wafer 1 and σ = 0.097 × 10^−3^ S/cm for crystal wafer 2) (Figure 5). The σ value for CLVO is lower than for Ca_9_Bi(VO_4_)_7_ ceramics (σ_bulk_ = 0.86 × 10^−3^ S/cm at 1200 K) [37]. The activation energy (E_a_) for the conductivity above the phase transition is the same for both directions (E_a_~1.2 eV). The conductivity in the framework of the CLVO structure results from the mobility of Ca^2+^ cations. Using the diffusion labeling method with the radioactive isotope ^45^Ca^2+^ (R = Eu^3+^) [38] and Tubandt method (*R* = La^3+^) [28], it has been shown that the conductivity in Ca_10.5–1.5*x*_*R_x_*(VO_4_)_7_ (*R* = La^3+^, Eu^3+^) solid solutions is provided by Ca^2+^ cations (r_VIII_ = 1.12 Å [39]). Larger cations, such as Pb^2+^ (r_VIII_ = 1.29 Å [39]), can also move in the β-TCP-type structure [40]. The β-TCP-type structure includes a 3D migration network for cations and is characterized by the 3D nature of ionic conductivity in accordance with bond valence energy landscape (BVEL) analysis [41]. The β-TCP-related framework contains three conductivity pathways; Ca^2+^ or Pb^2+^ cations can migrate along all pathways through the common faces of polyhedra or between faces of neighboring polyhedra. The *M*4 sites are involved in all pathways [40,41].

According to crystal structure refinement, the *M*5O_6_ octahedra in CLVO are occupied by Ca^2+^, while Ca^2+^ and La^3+^ cations occupy *M*1–*M*3 sites. The *M*4 and *M*6 sites are vacant. The occupancy of the *M*4 site in the β-TCP-related compounds can vary from 0 to 1. For example, the *M*4 site is half occupied by Ca^2+^ in the Ca_3_(VO_4_)_2_ structure [42], while it is completely occupied by K^+^ in Ca_10_K(VO_4_)_7_ [43]. The *M*6 site is always vacant in all known compounds with the β-TCP-type structure. Thus, the *M*4 and *M*6 sites are not structure-forming in comparison to the *M*1-*M*3 and *M*5 sites. However, they play an important role in the ferroelectric–paraelectric (FE-PE) phase transition [41] and take part in the ionic mobility of cations [40,41] within the β-TCP-type framework.

**Figure 8:**
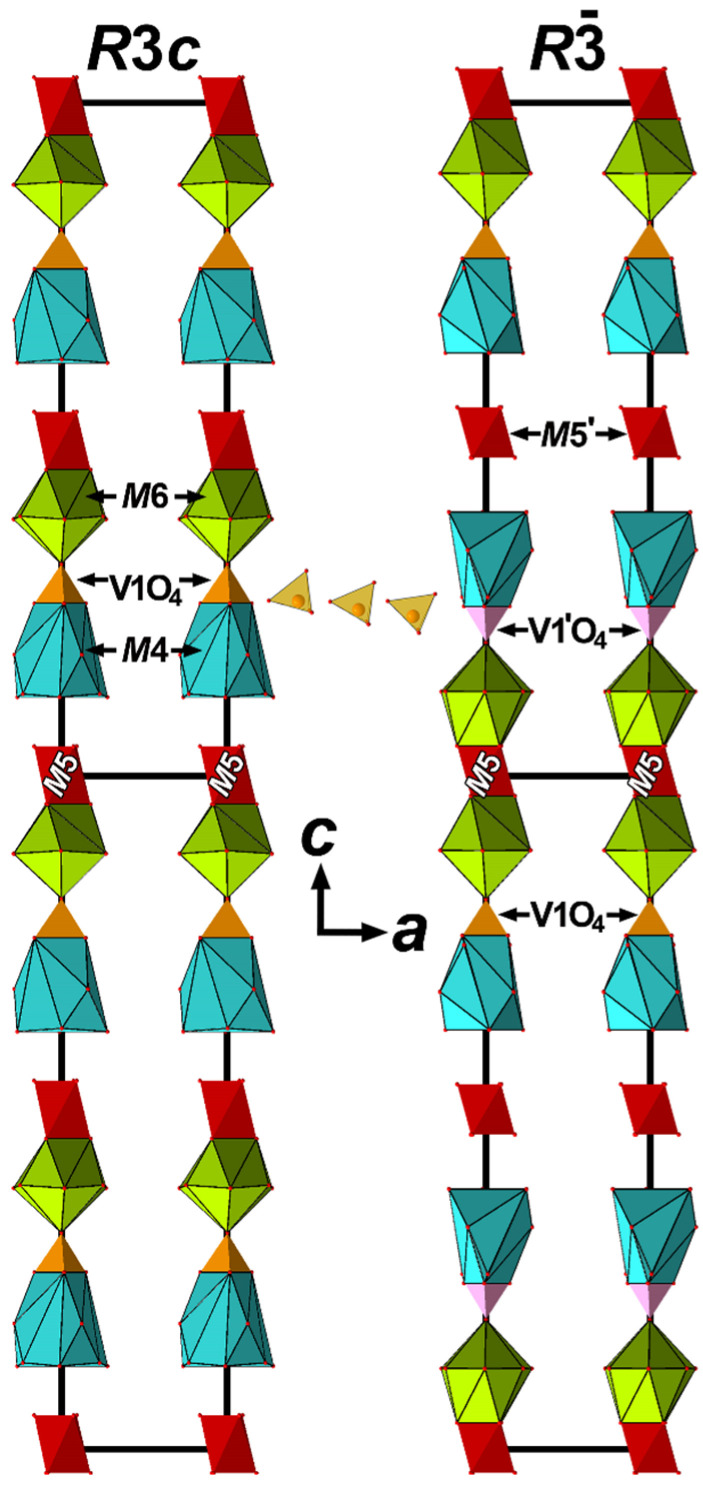
Alternation of VO_4_ tetrahedra, *M*5O_6_ octahedra, *M*4 and *M*6 sites polyhedra in the ferroelectric (left, SG *R*3*c*, cell parameter *c* doubled) and paraelectric (right, SG *R*$\bar{3}$) phases of the Ca_9_La(VO_4_)_7_ structure.

The crystal structure Ca_9_Fe(PO_4_)_7_ [34] were studied at 300 K (ferroelectric phase) and 973 K (paraelectric phase). Experimental data on the structures of the paraelectric phase of Ca_9_*R*(PO_4_)_7_ (*R* = Fe, In) were used to describe the structure of the paraelectric phase of CLVO. In the ferroelectric phase of CLVO, all V1O_4_ tetrahedra are oriented along the three-fold axis in the same direction (Figure 8, left). This orientation of V1O_4_ tetrahedra makes up the main contribution to the formation of the polar direction. Analysis of the CLVO structure showed cations in the *M*1 and *M*2 positions, and V2O_4_ and V3O_4_ tetrahedra deviate only slightly from the centrosymmetric arrangement, as in Ca_9_Fe(PO_4_)_7_ [34]. In CLVO, the *M*3 position is located close to the center of symmetry. Apparently, for this reason, the contribution of the cations in the *M*1, *M*2 and *M*3 positions and the V2O_4_ and V3O_4_ tetrahedra to the formation of the polar direction is much smaller than that of the V1O_4_ tetrahedra. During the ferroelectric ↔ parelectric transition or vice versa, the cations in the *M*1, *M*2, and *M*3 positions and the V2O_4_ and V3O_4_ tetrahedra should only shift slightly and become ordered upon heating or shift and become disordered upon cooling. Apparently, such small displacements are accompanied by an insignificant change in the SHG signal in the temperature range of 600–1100 K (Figure 1a). Since all V1O_4_ tetrahedra in the FE phase are oriented in the same direction, the transition to the PE phase should be accompanied by a rotation of half of the V1O_4_ tetrahedra, forming the opposite orientation. For the transition from the FE phase to the PE phase, it is sufficient for the V1O_4_ tetrahedra to rotate by 60° (Figure 8).

This rotation results in neighboring V1O_4_ tetrahedra on the 3-*fold* axis being linked by a center of symmetry (at the *M*5 site). This tetrahedron rotation is accompanied by a very small change in the thermal effect: Q~6.0 kJ/mol. After this rotation, three O2 oxygen atoms remain in their original positions, while half of the O1 oxygen atoms on the 3-*fold* axis move to another location (Figure 8). During the FE-PE phase transition, half of the *M*4 sites transform into *M*6 sites and vice versa (Figure 8).

X-ray diffraction cannot detect these structural changes [44] because the main difference is the repositioning of some O1 oxygen atoms along the 3-*fold* axis. Consequently, many studies have misdetermined the symmetry [45,46] of compounds with the β-TCP-type structure. Only the SHG method or dielectric spectroscopy can unambiguously determine whether compounds are centrosymmetric or non-centrosymmetric.

Near the phase transition temperature, when the V1O_4_ tetrahedron rotates, the SHG response drops sharply. This sharp drop occurs when half of the V1O_4_ tetrahedron rotates (Figure 8). It should also be noted that in CLVO, the *M*1 and *M*2 positions are unequally occupied by La^3+^ cations (Figure 7). For this reason, near the phase transition temperature, some La^3+^ cations must transform from the *M*1 position to the *M*2 position.

This transition equalizes the occupancies of these sites with Ca^2+^ or La^3+^ cations. In cases where the *M*1 and *M*2 positions are occupied by the same cations, the SHG signal value changes sharply only at the phase transition temperature, for example, Ca_9_Fe(PO_4_)_7_ [34].

At the T_R_ temperature, both centrosymmetric and non-centrosymmetric phases with a β-TCP-type structure exist [36,44]. Depending on the cation composition, the structure exhibits either FE [14,34,37] or AFE [36] properties. These properties are more characteristic of phosphates. However, vanadium-based compounds are always non-centrosymmetric at TR and transform into a centrosymmetric state as temperature increases. The centrosymmetric state appears at the T_R_ temperature only when some vanadium cations are replaced by phosphorus [47].

**Figure 9:**
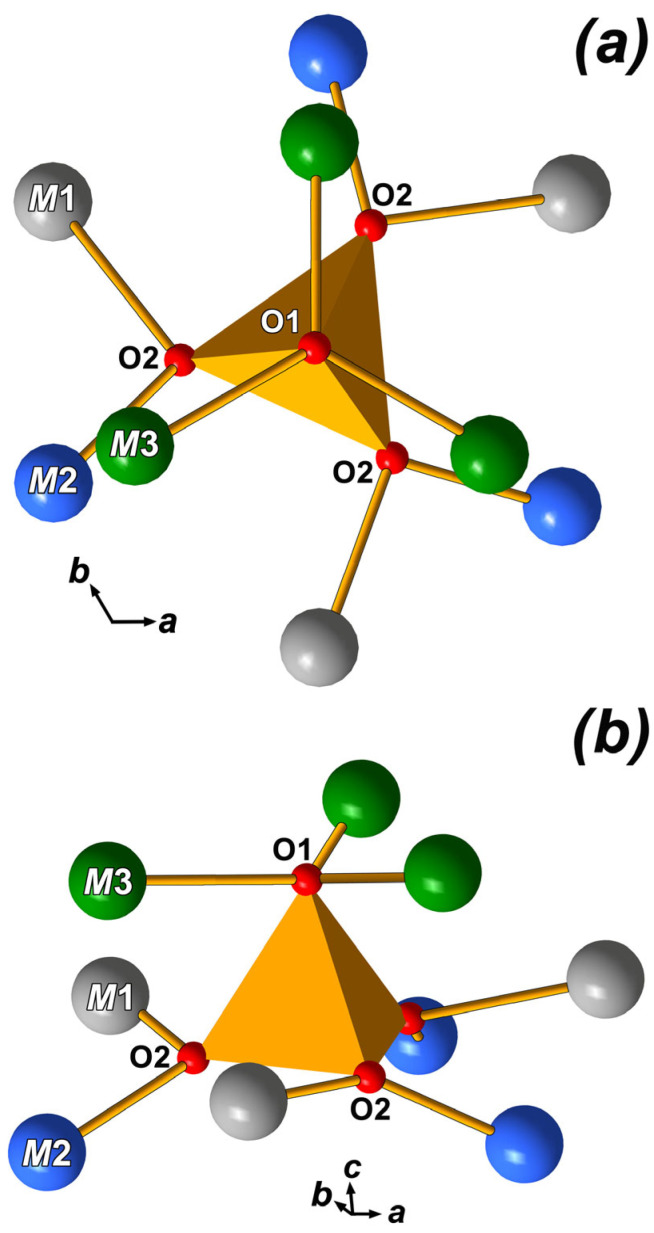
The surrounding of the V1O_4_ tetrahedra by positions *M*1, *M*2 and *M*3 in the Ca_9_La(VO_4_)_7_ structure: projections along (**a**) and perpendicular (**b**) the three-*fold* axis.

In CLVO, as in other isostructural compounds, the V1O1(O2)_3_ tetrahedra are strongly bonded to neighboring Ca^2+^ or La^3+^ cations located at the *M*1-*M*3 sites at distances of 2.524 Ǻ (*M*1-O2), 2.392 Ǻ (*M*2-O2), 2.921 Ǻ (*M*3-O2), and 2.575 Ǻ (3*M*3-O1) (Figure 9 and Table S3 of the Supplementary Materials). Each O2 oxygen atom is bonded to three cations in the *M*1, *M*2 and *M*3 sites, while the O1 oxygen atom binds three cations at the *M*3 site (Figure 7b). However, with increasing temperature, the *M*-O bonds break, and the V1O_4_ tetrahedra rotate by 60° (Figure 9). The phase transition temperature varies widely depending on cations present in the structure. Among the vanadates, Ca_3_(VO_4_)_2_ exhibits the highest FE-PE phase transition temperature (T_c_ = 1383 K) [48], while Ca_6_Pb_4.5_(VO_4_)_7_ shows the lowest one (T_c_ = 770 K) [42]. Phosphates consistently have lower phase transition temperatures than similar vanadates [49]. The *M*4 site occupancy significantly affects the phase transition temperature. When a cation occupies the *M*4 site, it hinders the rotation of the V1O_4_ tetrahedra (Figure 7). When Ca^2+^ is replaced by La^3+^ in CLVO according to the scheme 3Ca^2+^ = 2La^3+^ + ☐, vacancies form in the *M*4 site, and the phase transition temperature decreases significantly to T_c_ = 1224 K (ΔT = 159 degrees).

The occupancy of the *M*4 site significantly affects the laser characteristics of vanadate single crystals with the β-TCP structure. Single crystals with an unoccupied *M*4 position, such as Ca_9_La(VO_4_)_7_ [12,13], Ca_9_Yb(VO_4_)_7_ [14], and Ca_9_La(VO_4_)_7_:Nd^3+^ [23], exhibit noticeable scattering of laser radiation. In contrast, for single crystals with an occupied *M*4 position, for example, Nd:Ca_8.53_K_1.09_La_0.95_(VO_4_)_7_ [12] and Nd:Ca_9.03_Na_1.08_La_0.62_(VO_4_)_7_ [13], scattering is not observed. The scattering of laser radiation is associated with the formation of a domain structure, which is found in Ca_9_La(VO_4_)_7_ [50] (the *M*4 position is unoccupied) and not found in Ca_10_Li(VO_4_)_7_ [50] (the *M*4 position is occupied).

CLVO single crystals were grown at temperatures above 1773 K, which is significantly higher than the FE-PE phase transition temperature (T_c_ = 1224 K). During crystal growth, a centrosymmetric structure (SG *R*$\bar{3}$c or *R*$\bar{3}$m) forms. Upon cooling to T_c_ = 1224 K, a transition to a non-centrosymmetric (SG *R*3*c*) structure occurs. As shown above, this transformation involves the rotation of half of the V1O_4_ tetrahedra. In the CLVO structure, the empty *M*4 site imposes no restrictions on tetrahedron rotation in different parts of the crystal. Consequently, numerous domains form, scattering laser radiation. In Nd:Ca_8.53_K_1.09_La_0.95_(VO_4_)_7_ [12] and Nd:Ca_9.03_Na_1.08_La_0.62_(VO_4_)_7_ [13] single crystals, the *M*4 site is completely occupied by alkali metal cations. At T_R_ temperature, these phases crystallize in a trigonal non-centrosymmetric SG *R*3*c* [12,13]. Apparently, when single crystals with an occupied *M*4 site form at high temperatures, a non-centrosymmetric structure develops that remains stable upon cooling. No additional domains form, and therefore, these crystals do not scatter laser radiation.

CLVO serves as a versatile matrix for incorporating various rare earth elements and other cations. Vacancies in its structure enable both isovalent substitutions—replacing lanthanum with other rare earth elements or calcium with strontium or barium—and heterovalent substitutions (2Ca^2+^ = *RE*^3+^ + *Me*^+^), creating compounds with multifunctional properties. By introducing Nd^3+^, Er^3+^, Yb^3+^ or other cations into the CLVO lattice, it is possible to create self-frequency-doubling laser materials that operate in various spectral regions [12,13,14,23].

## 4. Materials and Methods

### Experimental Details

A CLVO single-crystal boule was grown using the Czochralski method. The Czochralski growth conditions were described previously [32].

Powder X-ray diffraction (PXRD) data for the milled CLVO single crystal were obtained on a Thermo ARL X’TRA powder diffractometer (Waltham, MA, USA, CuK_α_ radiation, λ = 1.5418 Å, Bragg–Brentano geometry, scintillation detector). PXRD data were collected in the 2θ range from 5 to 60° with a resolution of 0.02°. The X-ray diffraction pattern of the milled single-crystal matched literature data is given in the ICDD powder database (PDF2 46-0430). All reflections in the CLVO X-ray diffraction patterns are indexed in the *R*3*c* space group (SG), which is standard for the β-TCP-type structure.

The single-crystal X-ray data for CLVO were collected at room temperature (T_R_) on a Rigaku XtaLAB Synergy-S diffractometer (Akishima-shi, Japan) with MoK_α_ radiation (λ = 0.71073 Å) and a Hyrid Pixel Array detector using the *ω-θ* scanning mode. A colorless crystal (0.11 × 0.10 × 0.15 mm) of CLVO was used for single-crystal X-ray data collection. A total of 20,426 reflections found within the range of 3.73 ≤ *θ* ≤ 30.56°, and −12 ≤ *h* ≤ 15; −14 ≤ *k* ≤ 13; and −50 ≤ *l* ≤ 54 were measured. The initial model for CLVO structure refinement was based on the atomic coordinates of the same compound obtained using the Rietveld method [16]. CLVO structure refinement was carried out using the JANA2006 program packages [51]. Illustrations were produced with the JANA2006 program packages in combination with the DIAMOND program [52].

A Tescan VEGA3 scanning electron microscope (TESCAN, a.s., Brno, Czech Republic) was used to determine the chemical composition of CLVO by energy-dispersive X-ray (SEM-EDX) analysis. The Ca_K_, V_K_ and La_L_ lines in the EDX spectra were used. The oxygen and phosphorus contents were not quantified by EDX. The elemental composition of the CLVO single crystal corresponded to the specified stoichiometry (wt. %), theoretical/experimental: Ca(42.1/42.9 ± 0.5), La(16.2/16.5 ± 0.3), and V(41.6/40.5 ± 0.5).

The second optical harmonic generation study was carried out for the synthesized ceramic powders using the “reflection” scheme [53] on particles sized 400–500 µm. The SHG at the wavelength of λ_ω_ = 532 nm was excited by a Minilite-I YAG (Amplitude Laser Inc., Santa Clara, CA, USA): Nd-laser operating in a Q-switched mode at λ_ω_ = 1064 nm and registered in the reflection geometry. Polycrystalline α-SiO_2_ with 3–5 µm sized particles was used as a standard in order to calibrate the intensity of the SHG response *I*_2ω_ according to the q = *I*_2ω_/*I*_2ω_(SiO_2_) relationship. The part of the CLVO single crystal was previously milled into 3–5 µm powder for the sake of comparability with the reference α-SiO_2_ sample.

The SDT Q600 V8.1 thermal analyzer Build 99 (TA Instruments, New Castle, DE, USA) was used to perform differential scanning calorimetry (DSC). The studies were carried out in Pt crucibles in air at temperatures of 303–1300 K with a heating/cooling rate of 10 degrees/min.

The dielectric properties in the heating/cooling mode were measured on a Novocontrol Beta-N (Montabaur, Germany) setup with a “ProboStat” measurement cell using a four-wire two-contact method in the frequency range of 10–1 × 10^6^ Hz at 280–1300 K in air. The frequency dependences of the complex impedance Z*(ω) = Z’(ω) + iZ’’(ω) were measured during step heating under temperature stabilization conditions (the average heating rate was 2 K/min). The dielectric property measurements were performed on polished crystal wafers cut parallel (║) and perpendicular (┴) to the c axis (Figure 2). A gold paste was put on the flat parallel surfaces of the crystal wafer. After applying the paste, the sample was kept in a drying oven at 400 K for 20 min to dry. Additional annealing at 1025 ± 25 K for 1 h in air was performed to ensure good electrical contact between the sample and Au electrodes thus formed. Electrical conductivity (*σ*) was calculated from impedance spectra by means of equivalent electric circuits.

## 5. Conclusions

The crystal structure of CLVO grown using the Czochralski technique was refined using single-crystal data, and dielectric, nonlinear optical, and ion-conducting properties were studied. The distribution of Ca^2+^ and La^3+^ cations over structural sites was established and compared with the one refined previously using powder X-ray diffraction (PXRD) data. Dielectric spectroscopy and second-harmonic generation (SHG) methods revealed a reversible first-order ferroelectric phase transition at T_c_ = 1224 K on heating and 1215 K on cooling. This ferroelectric–paraelectric phase transition involves a complex structural rearrangement, including a 60° rotation of the V1O_4_ tetrahedron and minor displacements of calcium and lanthanum cations. The conductivity varies little across different crystal directions. The activation energy for conductivity above the phase transition is E_a_~1.2 eV for all directions. The influence of composition on the phase transition temperature, laser scattering, and the formation of ferroelectric and nonlinear optical properties is discussed. The causes of laser scattering in a CLVO single crystal activated with Nd^3+^, Er^3+^, and Yb^3+^ cations are substantiated.

## Figures and Tables

**Table 1:** Crystallographic data for Ca_9_La(VO_4_)_7_.

Crystal Data		
Chemical formula	Ca_9_La(VO_4_)_7_	
Formula weight	1303.2	
Temperature (K)	293	
Crystal system	Trigonal	
Space group (SG)	*R*3*c*	
Lattice parameters:		
*a* (Å)	10.9018(2)	
*c* (Å)	38.1593(6)	
*V* (Å^3^)	3927.60(12)	
*Z*	6	
Calculated density, Dx (g·cm^−3^)	3.31	
Color	Colorless	
Crystal size (mm)	0.11 × 0.10 × 0.15	
**Data collection and Refinement**		
**Diffractometer**	Crystal Data	
Radiation type/wavelength (λ, Å)	Mo K_α_/0.71073	
Radiation monochromator	Mirror	
*θ* range (o)	3.73–30.56	
*hkl* ranges	−12 < *h* < 15; −14 < *k* < 13; −50 < l < 54	
Total number of reflections	17,339	
Criteria of observation	I > 3σ(I)	
Number of averaged reflections (observed/all)	1197/1257	
No. of refined parameters	139	
GOF (observed/all)	1.14/1.15	
R and Rw, % (Robs/Rall)	1.62/1.78 and 1.82/1.84	
Max./min. residual electron density (*e* × Å^−3^)	0.32/−0.61	
CSD No. 2524264		

**Table 2:** Comparison of the lattice parameters, cation site occupancies, selected distances (Å), polyhedra distortion index (DI) and tetrahedral distortion parameters ∆d and ∆α for Ca_9_La(VO_4_)_7_ structure refined from single-crystal and powder data.

		Single Crystal	Powder [16]
Lattice Parameters:		This Work	
*a*, Å *c*, Å		10.9018(2) 38.1593(6)	10.8987(5) 38.147(1)
V1O_4_	*<*V1-O>	1.714	1.69
	∆*d*	0.00001	0.0008
	∆α	0.0017	0.0038
V2O_4_	*<*V2-O>	1.711	1.72
	∆*d*	0.00012	0.00028
	∆α	0.0027	0.0041
V3O_4_	*<*V3-O>	1.701	1.71
	∆*d*	0.00002	0.00056
	∆α	0.0013	0.0013
Occupancy *M*1		0.940(2)Ca^2+^+ 0.060(2)La^3+^	0.91(1)Ca^2+^+ 0.09(1)La^3+^
<*M*1-O>		2.458	2.44
DI(*M*1-O)		0.0283	0.0391
Occupancy *M*2		0.965Ca^2+^+ 0.035La^3+^	0.98(1)Ca^2+^+ 0.02(1)La^3+^
<*M*2-O>		2.529	2.54
DI(*M*2-O)		0.0675	0.0678
Occupancy *M*3		0.762(1)Ca^2+^+ 0.238(1)La^3+^	0.78(1)Ca^2+^+ 0.22(1)La^3+^
<*M*3-O>		2.631	2.63
DI(*M*3-O)		0.0476	0.0439
Occupancy *M*5		Ca^2+^	Ca^2+^
<*M*5-O>		2.313	2.31
DI(*M*5-O)		0.0067	0.0303

## Data Availability

The research data are available upon official, reasonable request.

## Supplementary Materials

The following supporting information can be downloaded at https://www.mdpi.com/article/10.3390/molecules31060984/s1, Table S1: Fractional atomic coordinates and isotropic atomic displacement parameters (U_iso_) for Ca_9_La(VO_4_)_7_; Table S2: Anisotropic atomic displacement parameters in Ca_9_La(VO_4_)_7_; Table S3: Selected interatomic distances (Å) and angles in the [VO_4_]^3−^ tetrahedra for Ca_9_La(VO_4_)_7_.
