# Supplementary material for: Temperature Evolution of Crystal Structure, Ferroelectricity and Ionic Conductivity of Ca9La(VO4)7
Source: Molecules. 2026 Mar 15;31(6):984. doi: 10.3390/molecules31060984 (PMC13029712; doi:10.3390/molecules31060984)
Supplement: Supplementary file 1 [file molecules-31-00984-s001.zip › molecules-4147222-supplementary.pdf]

Supplementary Materials

Temperature Evolution of Crystal Structure, Ferroelectricity and Ionic Conductivity of $Ca_9La(VO_4)_7$

Oksana V. Baryshnikova, Bogdan I. Lazoryak, Vladimir A. Morozov, Sergey Yu. Stefanovich, Alexander V. Mosunov, Eldar M. Gallyamov, Sergey M. Aksenov and Dina V. Deyneko

Table S1. Fractional atomic coordinates and isotropic atomic displacement parameters (U_{iso}) for for $Ca_9La(VO_4)_7$.

Atom	Occupancy	*x*	*y*	*z*	U_{iso}*100
*M*1	0.940(2)Ca^{2+}+ 0.060(2)La^{3+}	0.72537(5)	0.86100(5)	0.43097(3)	1.14(2)
*M*2	0.965Ca^{2+}+ 0.035La^{3+}	0.61342(6)	0.82292(5)	0.23341(3)	1.27(2)
*M*3	0.762(1)Ca^{2+}+ 0.238(1)La^{3+}	0.12326(4)	0.26843(4)	0.32431(3)	1.57(2)
*M*5	1Ca^{2+}	0	0	0	1.87(2)
V1	1V	0	0	0.26772(4)	1.03(2)
V2	1V	0.68426(5)	0.85863(5)	0.13405(3)	0.91(2)
V3	1V	0.65417(5)	0.84759(5)	0.03224(3)	0.79(2)
O1	1O	0	0	0.3128(1)	2.0(1)
O2	1O	0.0129(2)	0.8551(2)	0.25628(7)	1.85(8)
O3	1O	0.7078(3)	0.9052(4)	0.17665(7)	3.6(2)
O4	1O	0.7679(2)	0.7688(2)	0.12184(7)	1.98(9)
O5	1O	0.7237(2)	0.0133(2)	0.11202(6)	1.22(7)
O6	1O	0.5074(2)	0.7514(2)	0.12463(8)	1.88(8)
O7	1O	0.5929(2)	0.9585(2)	0.04369(7)	1.83(9)
O8	1O	0.5718(3)	0.6851(3)	0.05079(7)	2.67(9)
O9	1O	0.8288(2)	0.9269(2)	0.04377(7)	1.42(8)
O10	1O	0.6272(3)	0.8214(2)	0.98837(7)	1.83(9)

Table S2. Anisotropic atomic displacement parameters in $Ca_9La(VO_4)_7$.

	U^{11}	U^{22}	U^{33}	U^{12}	U^{13}	U^{23}
*M*1	0.0119(3)	0.0126(3)	0.0105(3)	0.0068(2)	-0.0002(2)	-0.0016(2)
*M*2	0.0134(3)	0.0117(3)	0.0118(3)	0.0054(2)	-0.0034(2)	-0.0014(2)
*M*3	0.0122(2)	0.0208(2)	0.0137(2)	0.00785(15)	-0.0008(1)	0.0053(1)
*M*5	0.0244(3)	0.0244(3)	0.0072(4)	0.0122(2)	0	0
V1	0.0094(2)	0.0094(2)	0.0123(4)	0.0047(1)		
V2	0.0105(2)	0.0099(2)	0.0084(2)	0.0063(2)	0.0026(2)	0.0017(2)
V3	0.0073(2)	0.0087(2)	0.0068(2)	0.0034(2)	0.0000(2)	0.0000(2)
O1	0.0235(12)	0.0235(12)	0.0134(17)	0.0118(6)	0	0
O2	0.0158(10)	0.0126(9)	0.0288(11)	0.0083(8)	0.0085(8)	0.0025(8)
O3	0.0547(17)	0.059(2)	0.0140(11)	0.0438(17)	0.0005(11)	0.0003(11)
O4	0.0255(11)	0.0252(11)	0.0188(10)	0.0202(10)	0.0066(8)	0.0049(9)
O5	0.0108(9)	0.0093(9)	0.0144(9)	0.0035(7)	0.0018(7)	0.0020(7)
O6	0.0123(11)	0.0097(9)	0.0323(12)	0.0039(8)	0.0044(9)	0.0009(8)
O7	0.0237(11)	0.0271(11)	0.0143(9)	0.0205(9)	-0.0026(8)	-0.0053(9)
O8	0.0330(12)	0.0157(11)	0.0218(11)	0.0050(9)	0.008(1)	0.0077(9)
O9	0.0107(9)	0.0138(9)	0.0192(10)	0.0068(8)	-0.0034(8)	-0.0042(8)
O10	0.0261(12)	0.0232(11)	0.0089(10)	0.0147(9)	-0.0021(9)	-0.0018(8)

Table S3. Selected interatomic distances (Å) and angles in the $[VO_4]^{3-}$ tetrahedra for $Ca_9La(VO_4)_7$.

Bond	Distance, Å	Bond	Distance, Å
*M*1-O2	2.524(2)	*M*2-O2	2.392(3)
O5	2.540(3)	O3	2.373(3)
O6	2.528(2)	O4	2.414(3)
O6	2.461(4)	O5	2.364(3)
O7	2.418(3)	O7	2.881(3)
O8	2.318(4)	O8	2.860(3)
O10	2.379(3)	O9	2.449(2)
		O9	2.501(3)
mean	2.458	*mean*	2.529
DI	0.0283	DI	0.0675
*M*3-O1	2.575(9)	*M*5-O6×3	2.297(3)
O2	2.921(3)	O9×3	2.328(2)
O3	2.843(6)	*mean*	2.313
O4	2.506(3)	DI	0.0067
O5	2.410(3)		
O7	2.437(3)	V1-O1	1.722(4)
O8	2.657(3)	O2×3	1.711(3)
O10	2.628(2)	*mean*	1.714
O10	2.704(3)		
mean	2.631		
DI	0.0476		
V2-O3	1.684(3)	V3-O7	1.705(3)
O4	1.704(3)	O8	1.689(3)
O5	1.735(2)	O9	1.709(2)
O6	1.720(2)	O10	1.699(3)
mean	1.711	*mean*	1.701

Angle	Angle, °	Angle	Angle, °
O1-V1-O2×3	104.78(10)	O3-V2-O4	113.13(19)
O2-V1-O2×3	113.72(14)	O3-V2-O5	104.37(16)
		O3-V2-O6	110.90(15)
O7-V3-O8	116.41(14)	O4-V2-O5	118.85(13)
O7-V3-O9	107.92(11)	O4-V2-O6	107.00(12)
O7-V3-O10	106.00(15)	O5-V2-O6	102.03(12)
O8-V3-O9	107.25(14)		
O8-V3-O10	106.24(12)		
O9-V3-O10	113.20(14)		
